# Mechanisms of Dihydroartemisinin and Dihydroartemisinin/Holotransferrin Cytotoxicity in T-Cell Lymphoma Cells

**DOI:** 10.1371/journal.pone.0137331

**Published:** 2015-10-26

**Authors:** Qiuyan Wang, Shaoling Wu, Xindong Zhao, Chunting Zhao, Hongguo Zhao, Lanfen Huo

**Affiliations:** 1 Department of Hematology, Affiliated hospital, Qingdao University, Shandong, P.R. China; 2 Department of Hematology, Medical College of Qingdao University, Qingdao, Shandong, P.R. China; Georgetown University, UNITED STATES

## Abstract

The validated therapeutic effects of dihydroartemisinin (DHA) in solid tumors have encouraged us to explore its potential in treating T-cell lymphoma. We found that Jurkat cells (a T-cell lyphoma cell line) were sensitive to DHA treatment with a IC50 of dihydroartemisinin. The cytotoxic effect of DHA in Jurkat cells showed a dose- and time- dependent manner. Interestingly, the cytotoxic effect of DHA was further enhanced by holotransferrin (HTF) due to the high expression of transferrin receptors in T-cell lymphoma. Mechanistically, DHA significantly increased the production of intracellular reactive oxygen species, which led to cell cycle arrest and apoptosis. The DHA treatment also inhibited the expression of protumorgenic factors including VEGF and telomerase catalytic subunit. Our results have proved the therapeutic effect of DHA in T-cell lymphoma. Especially in combination with HTF, DHA may provide a novel efficient approach in combating the deadly disease.

## Introduction

Lymphoma is one of the most common malignant tumors of the hematological system. Furthermore, the incidence of lymphoma is increasing across multiple age brackets. Current treatment options for lymphoma include combination radiotherapy and chemotherapy, biological therapy, or hematopoietic stem cell transplantation. Despite advances in radiotherapy techniques and improved chemotherapy regimens, the 5-year survival rate for non-Hodgkin lymphoma is still low at approximately 69% [1], and the cure rate for T-cell lymphoma remains relatively poor. Novel strategies to improve the cure rate of patients with T-cell lymphoma are therefore urgently required.

Dihydroartemisinin (DHA) is the most active derivative of artemisinin and is isolated from the traditional Chinese herb *Artemisia annua L*. DHA possesses a potent anti-malarial effect, and recent studies have revealed a cytotoxic effect of DHA on several malignant tumor cell lines including those derived from ovarian, pancreatic, hepatocellular, and breast cancers [2–6]. This effect is likely mediated by an endoperoxide-bridge within the DHA molecule that facilitates production of free radicals or reactive intermediates after reacting with ferrous atoms [7,8], ultimately causing damage to biological macromolecules [9]. Artemisinin is activated by intracellular iron [10], and combined exposure to holotransferrin (HTF) and DHA can cause rapid death of leukemic cells [11]. Therefore, we speculated that DHA and HTF in combination could effectively target T-cell lymphoma cells. However, few studies to date have comprehensively assessed the cytotoxic mechanisms induced by DHA or DHA/HTF in T-cell lymphoma cells, and little is known regarding the antineoplastic potential of these drugs in T-cell lymphoma.

The cytotoxic mechanisms of DHA may be related to one or more of its previously demonstrated effects in solid tumors, which include regulation of angiogenesis, telomerase, cell apoptosis, cell cycle, reactive oxygen species (ROS), and the transferrin receptor (TfR). Artemisinin has anti-angiogenic activity that involves the generation of free radicals [12]. Vascular endothelial growth factor (VEGF) stimulates angiogenesis and its expression by tumor cells is closely related to tumor growth. Thus, the anti-angiogenic effects of DHA and DHA/HTF on T-cell lymphoma cells can be evaluated by measurement of VEGF mRNA expression. Telomerase activity is required for the development of most cancers [13, 14], and hematological tumors generally exhibit telomerase activity. The level of telomerase activity has important clinical and prognostic significance [15]. As the expression of human telomerase catalytic subunit (hTERT) correlates with telomerase activity [16], telomerase activity may be evaluated indirectly by measurement of hTERT mRNA expression. Most cancer cells possess elevated levels of TfR on the cell surface and have a high iron intake [17–21]. This high intracellular iron concentration may facilitate ROS generation in T-cell lymphoma cells following exposure to DHA/HTF.

Here we investigated the antineoplastic potential of DHA and DHA/HTF in human T-cell lymphoma cells and determined the mechanisms underlying this effect. ROS generation, angiogenesis, telomerase activity, apoptosis, and the cell cycle were assessed following treatment of T-cell lymphoma cells with DHA or DHA/HTF.

## Materials and Methods

### Materials and cell culture

DHA was purchased from Chunyou Biological Technology Corporation (Shanghai, China) and HTF was obtained from Boaosen Biological Technology Corporation (Beijing, China). DHA was stored as a stock solution of 8000 μM in dimethyl sulfoxide (DMSO; Sigma, California, USA) and at −20°C. The final concentration of DMSO in the culture medium was less than 0.1%. HTF was dissolved in ultrapure water at 4000 nM and stored at 4°C. DHA and HTF were freshly prepared for each experiment by diluting stock solutions in RPMI1640 medium.

Jurkat cells were used as a human T-cell lymphoma model and were purchased from the cell bank of the Chinese Academy of Sciences. Jurkat cells were cultured in RPMI1640 medium (HyClone, Beijing, China) containing 10% fetal bovine serum (Gibco, California, USA), 100 U/mL penicillin, and 100 μg/mL streptomycin and incubated at 37°C in a 5% CO_2_ humidified incubator. Cells at logarithmic growth phase were used for experiments.

### Cell viability assay

Jurkat cells (1 × 10^4^/well, in 100 μL culture medium) were seeded in 96-well plates (Corning Costar, Suzhou, China). The stock DHA solution was diluted in RPMI1640 medium to a final concentration of 2.5, 5, 10, 20, 40, or 80 μM. Triplicate wells were established for each condition, and cells were incubated for 24, 48, or 72 h, followed by addition of Cell Counting kit-8 (CCK-8) solution (Dojindo, Kyushu, Japan; 10 μL/well) and further incubation for 4 h. Sample absorbance was then read at 450 nm using a microplate absorbance reader (Sunrise, TECAN, Switzerland). DHA/HTF-treated cells were exposed to HTF at a final concentration of 20 nM and incubated for 1 h prior to addition of freshly prepared DHA. Three experimental groups were used: control (cells not exposed to drugs); DHA-treated cells (exposed to 10, 20, 40, or 80 μM DHA); and DHA/HTF-treated cells (exposed to 20 nM HTF for 1 h followed by addition of 20 μM DHA).

### Measurement of ROS production

Jurkat cells (1 × 10^6^/well) were seeded in 6-well plates (Corning Costar, Suzhou, China), treated with indicated concentrations of DHA or DHA/HTF, and cultured for 48 h at 37°C with 5% CO_2_ in a humidified incubator. Cells were collected by centrifugation at 800 g for 5 min at room temperature, resuspended with 10 μM diluted dichloro-dihydro-fluorescein diacetate (DCFH-DA; Beyotime Institute of Biotechnology, Jiangsu, China), and incubated for 20 min with mixing every 3–5 min. Cells were then washed three times with phosphate-buffered saline (PBS). Absorbance was determined using a multifunctional microplate absorbance reader (Infinite^®^ M1000, TECAN). The blank control contained 10 μM DCFH-DA and no cells, while positive control cells were treated for 15 min with Rosup (50 mg/mL, 1 μL) (Beyotime Institute of Biotechnology, JiangSu, China) before addition of DCFH-DA. Each condition was prepared in triplicate.

### Cell apoptosis analysis

Cellular apoptosis was assessed by Annexin V-FITC & propidium iodide (PI) staining (Annexin V/PI, BD Biosciences, New York, USA). Jurkat cells were treated as described above. Collected cells were washed with ice-cold PBS and centrifuged at 800 g for 5 min at room temperature. The supernatant was discarded and the cells resuspended in 300 μL 1× Binding Buffer, followed by addition of 5 μL Annexin V-FITC and incubation for 15 min at room temperature in the dark. After incubation, 5 μL PI working solution was added before an additional 5 min room temperature incubation. Finally, 200 μL 1× Binding Buffer was added. Each sample was analyzed by flow cytometry (FACS Calibur, BD Biosciences, New York, USA).

### Cell cycle analysis

Cell cycle was assessed using a cell cycle analysis kit (Beyotime Institute of Biotechnology, Jiangsu, China) and standard PI staining methods. Briefly, PI combined with double-stranded DNA produce a fluorescence signal that is proportional to the DNA content. The cell cycle can be analyzed according to the distribution of DNA content. The cell cycle can be analyzed according to the distribution of DNA content. Jurkat cells were treated as above. Collected cells were resuspended in 1 mL ice-cold PBS, transferred to 1.5 mL Eppendorf tubes, fixed with ice-cold 70% alcohol at 4°C overnight, washed with ice-cold PBS, resuspended in PI staining solution, and incubated for 30 min at 37°C. The numbers of cells at each phase of the cell cycle were measured using a flow cytometer (FACS Calibur, BD Biosciences, New York, USA).

### Isolation of RNA and RT-PCR

Total RNA was isolated using Trizol reagent (TaKaRa, Tokyo, Japan), and the quality and quantity of RNA were determined by spectrophotometry (Dynamica, UK). RNA was reverse transcribed using the Prime Script™ RT reagent kit (Perfect Real Time; TaKaRa, Tokyo, Japan) according to the manufacturer’s instructions. cDNA was then amplified by RT-PCR using the primers TfR: 5ʹ-TCTGACACGTCTGCCTACCCATTCG-3ʹ (forward) and 5ʹ-TATGATGGTTCACTCACGGAGCTTCG-3ʹ (reverse) [22]; VEGF: 5ʹ-ATGACGAGGGCCTGGAGTGTG-3ʹ (forward) and 5ʹ-CCTATGTGCTGGCCTTGGTGAG-3ʹ (reverse) [23]; and GAPDH: 5ʹ-AGAAGGCTGGGGCTCATTTG-3ʹ (forward) and 5ʹ-AGGGGCCATCCACAGTCTTC-3ʹ (reverse). Primers were synthesized by Sangon Biotech (Shanghai, China). Quantitative RT-PCR was performed using a CFX96 spectrofluorometric thermal cycler (Bio-Rad, USA) and SYBR^®^ Premix Ex Taq II (TaKaRa). PCR cycling conditions included an initial denaturation step at 95°C for 30 s and then 40 cycles of 95°C for 5 s and 60°C for 30 s. The 2^−△△CT^ method was used to calculate gene expression changes relative to control samples [24]. PCR amplification products were separated by 2% agarose gel electrophoresis, and visualized using a gel imaging system (Gel Doc™ XR, Bio-Rad, USA) to verify product size.

### Telomerase activity analysis

RNA extraction and reverse transcription was conducted as described above. Telomerase activity was assessed using a similar method to that of the Keygen real-time fluorescent quantitative PCR kit for telomerase (Keygen Biotech, Nanjing, China), whereby hTERT mRNA expression is measured as an indirect reflection of telomerase activity. PCR primers for hTERT were 5ʹ-TGGCTGATGAGTGTACGT-3ʹ (forward) and 5ʹ-TGTCTGATTCCAATGCTTTG-3ʹ (reverse). PCR cycling conditions and analysis were the same as those described above.

### Statistical analysis

Experimental data were analyzed by using Graphpad Prism 5.01, and expressed as mean ± standard deviation. Tests were carried out on the normal distribution of the data set that obtained from different groups and compared by repeated measures ANOVA. With *P*< 0.05 was considered statistically significant difference.

## Results

### Treatment with DHA and DHA/HTF decreased Jurkat cell viability

**Fig 1:**
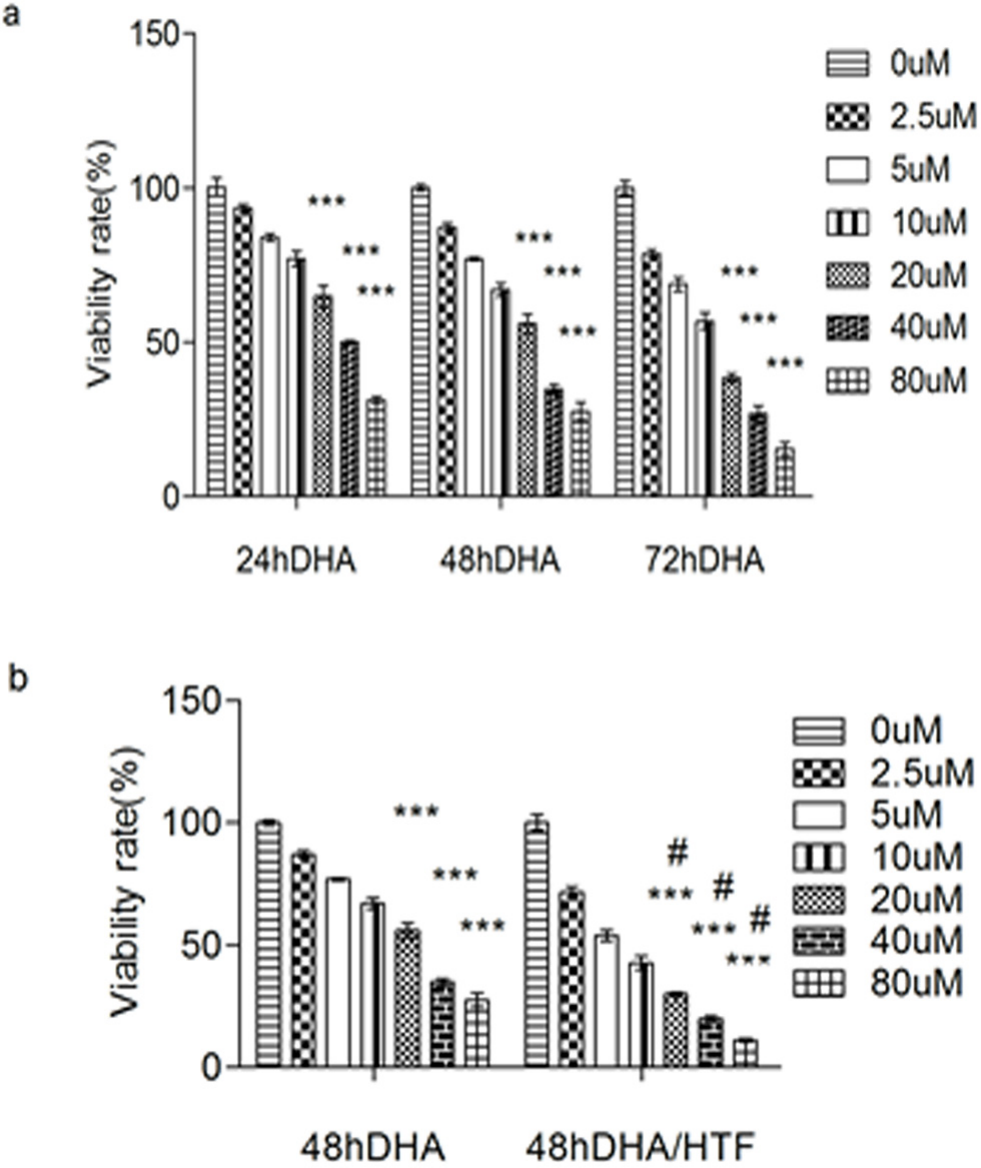
Treatment with DHA and DHA/HTF decreased Jurkat cell viability. Jurkat cells (1 × 10^4^/well) were exposed to DHA (0, 2.5, 5, 10, 20, 40, 80 μM) for 24, 48 or 72 h(a) or DHA/HTF for 48 h(b). The cell viability was assessed by CCK-8 assay. The results represented mean ± standard deviation. ***p<0.001, compared with the control group, **#** p<0.001, compared with DHA.

We first exposed Jurkat cells to DHA (0, 2.5, 5, 10, 20, 40, or 80 μM) for 24, 48, or 72 h or DHA/HTF for 48 h and assessed cell viability by CCK-8 assay. DHA treatment reduced cell viability in a dose- and time-dependent manner (Fig 1A). IC50 values for DHA were 90.66, 21.73, and 14.56 μM for treatments of 24, 48, and 72 h, respectively. Furthermore, cytotoxicity was increased by treatment with DHA/HTF compared with DHA alone (p < 0.001; Fig 1B). Combined treatment with 20 μM HTF reduced the DHA IC50 value to 6.33 μM and the cell inhibition rate was 3.4 times lower than for 20 μM DHA treatment (p < 0.001).

### HTF enhanced ROS production following DHA exposure

**Fig 2:**
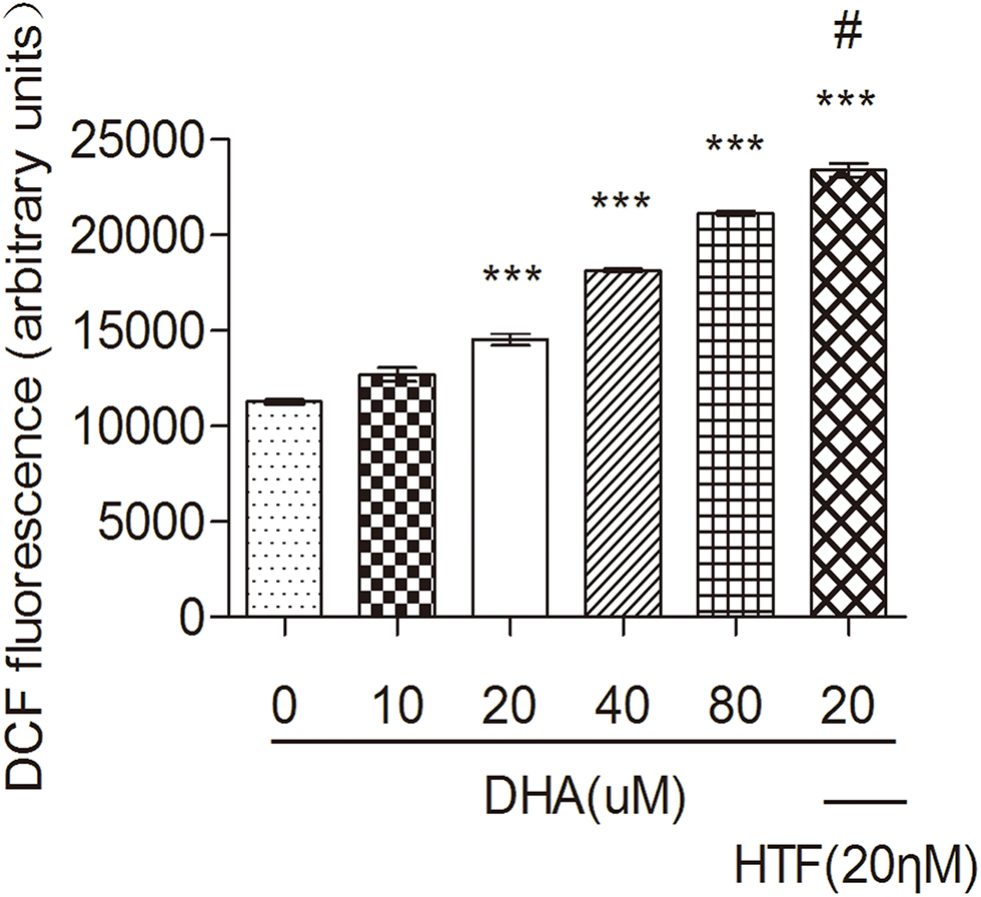
HTF enhanced ROS production following DHA exposure. Jurkat cells (1 × 10^6^/well) were exposed to DHA (0, 10, 20, 40, 80 μM) or DHA/HTF for 48 h. The fluorescent probe DCFH-DA identified a dose-dependent increase in ROS following exposure to DHA. Furthermore, combined exposure of Jurkat cells to 20 μM DHA and 20 μM HTF produced over three-fold more ROS than exposure to 20 μM DHA alone (p < 0.001). The results represented mean ± standard deviation. ***p<0.001, compared with the control group, **#** p<0.001, compared with DHA.

We next exposed Jurkat cells to DHA (0, 10, 20, 40, or 80 μM) or DHA/HTF for 48 h. The fluorescent probe DCFH-DA identified a dose-dependent increase in ROS following exposure to DHA (Fig 2). Furthermore, combined exposure of Jurkat cells to 20 μM DHA and 20 μM HTF produced over three-fold more ROS than exposure to 20 μM DHA alone (p < 0.001).

### DHA and DHA/HTF promoted Jurkat cell apoptosis

**Fig 3:**
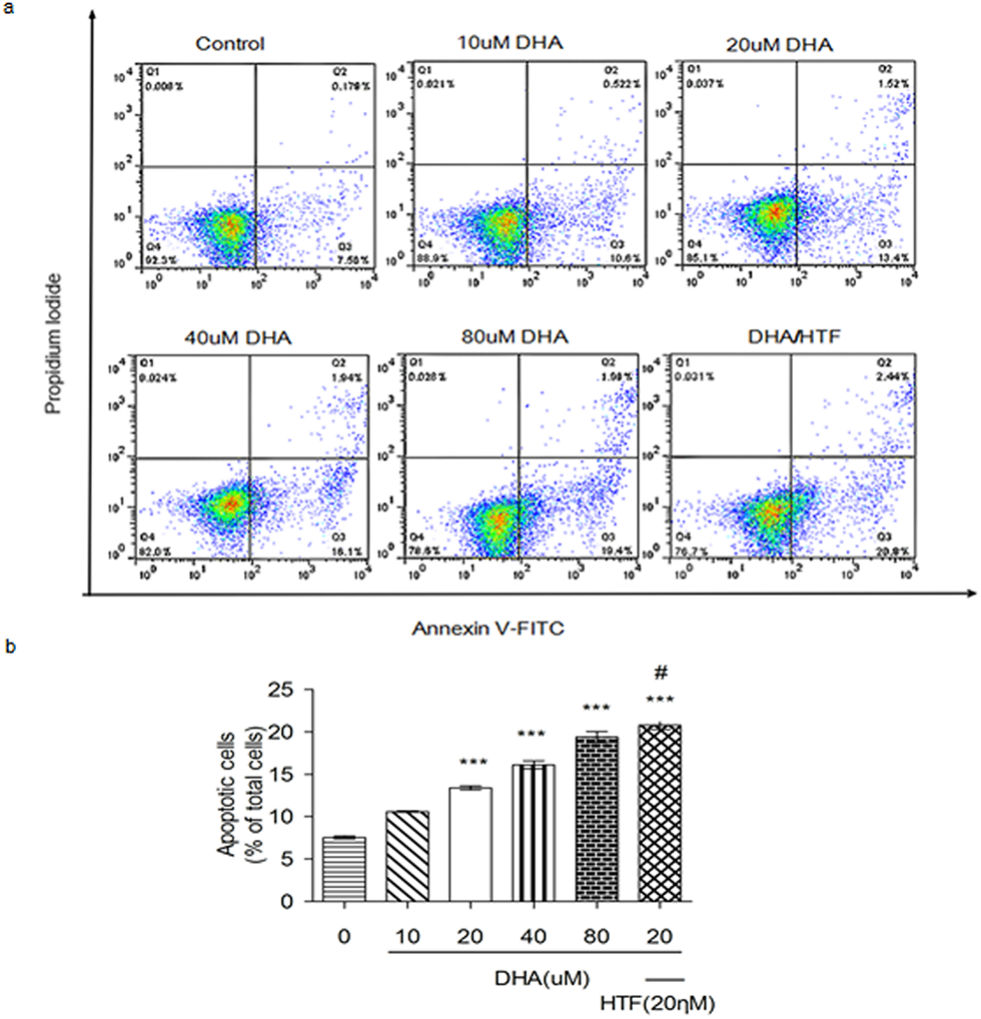
DHA and DHA/HTF promote Jurkat cell apoptosis. Jurkat cells (1 × 10^6^/well) were exposed to DHA (0, 10, 20, 40, 80 μM) or DHA/HTF for 48 h, and measured apoptosis by Annexin V/PI staining and flow cytometry. DHA induced a dose-dependent increase in apoptosis (a). This difference was statistically significant (p < 0.001). DHA/HTF induced a 2.75-fold increase in apoptosis relative to control (p < 0.001). The results represented mean ± standard deviation (b). ***p<0.001, compared with the control group, **#** p<0.001, compared with DHA.

We exposed Jurkat cells to DHA (0, 10, 20, 40, or 80 μM) or DHA/HTF for 48 h and measured apoptosis by Annexin V/PI staining and flow cytometry. DHA induced a dose-dependent increase in apoptosis (Fig 3B). Apoptosis rates in cells treated with DHA (20 μM), DHA/HTF were 13.4% and 20.8%, respectively, and this difference was statistically significant (p < 0.001). DHA/HTF induced a 2.75-fold increase in apoptosis relative to the control (p < 0.001).

### DHA and DHA/HTF impaired cell cycle progression in Jurkat cells

**Fig 4:**
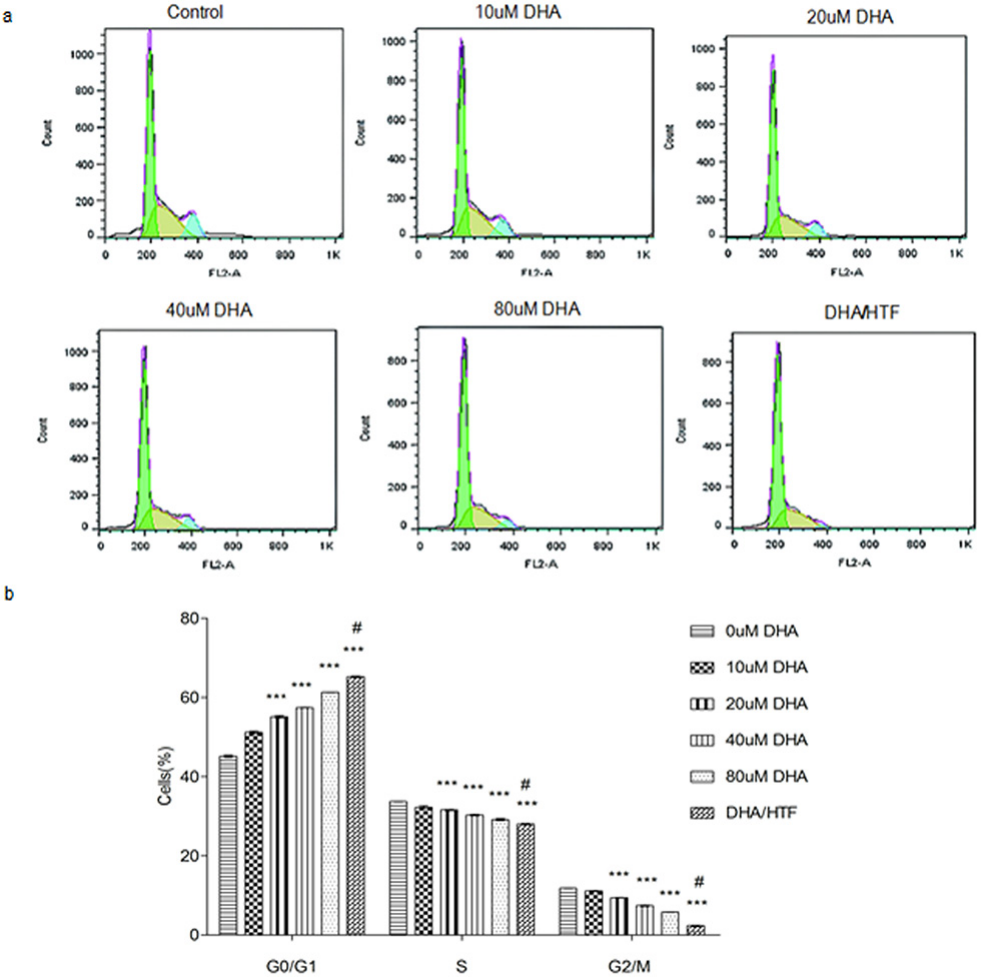
DHA and DHA/HTF impaired cell cycle progression in Jurkat cells. Jurkat cells (1 × 10^6^/well) were exposed to DHA (0, 10, 20, 40, 80 μM) or DHA/HTF for 48 h, Cell cycle analysis of DHA- and DHA/HTF-treated cells was performed using flow cytometry. The ratio of cells in S and G2/M phases decreased while the numbers in G0/G1 phase increased in a dose-dependent manner (a, b). The results represented mean ± standard deviation. ***p<0.001, compared with the control group, **# p**<0.001, compared with DHA.

Cell cycle analysis of DHA- and DHA/HTF-treated cells was performed using standard flow cytometry and PI staining techniques. Following treatment with DHA or DHA/HTF, the ratio of cells in S and G2/M phases decreased while the numbers of cells in G0/G1 phase increased in a dose-dependent manner (Fig 4A and 4B.). Treatment with DHA/HTF increased the ratio of cells in G0/G1 by 44.5% and decreased the number of cells in S and G2/M phases by 16.9% and 80.3%, respectively. These results indicate that DHA inhibited Jurkat cells at the G0/G1 phase.

### Treatment of Jurkat cells with DHA or DHA/HTF inhibited TfR mRNA expression

**Fig 5:**
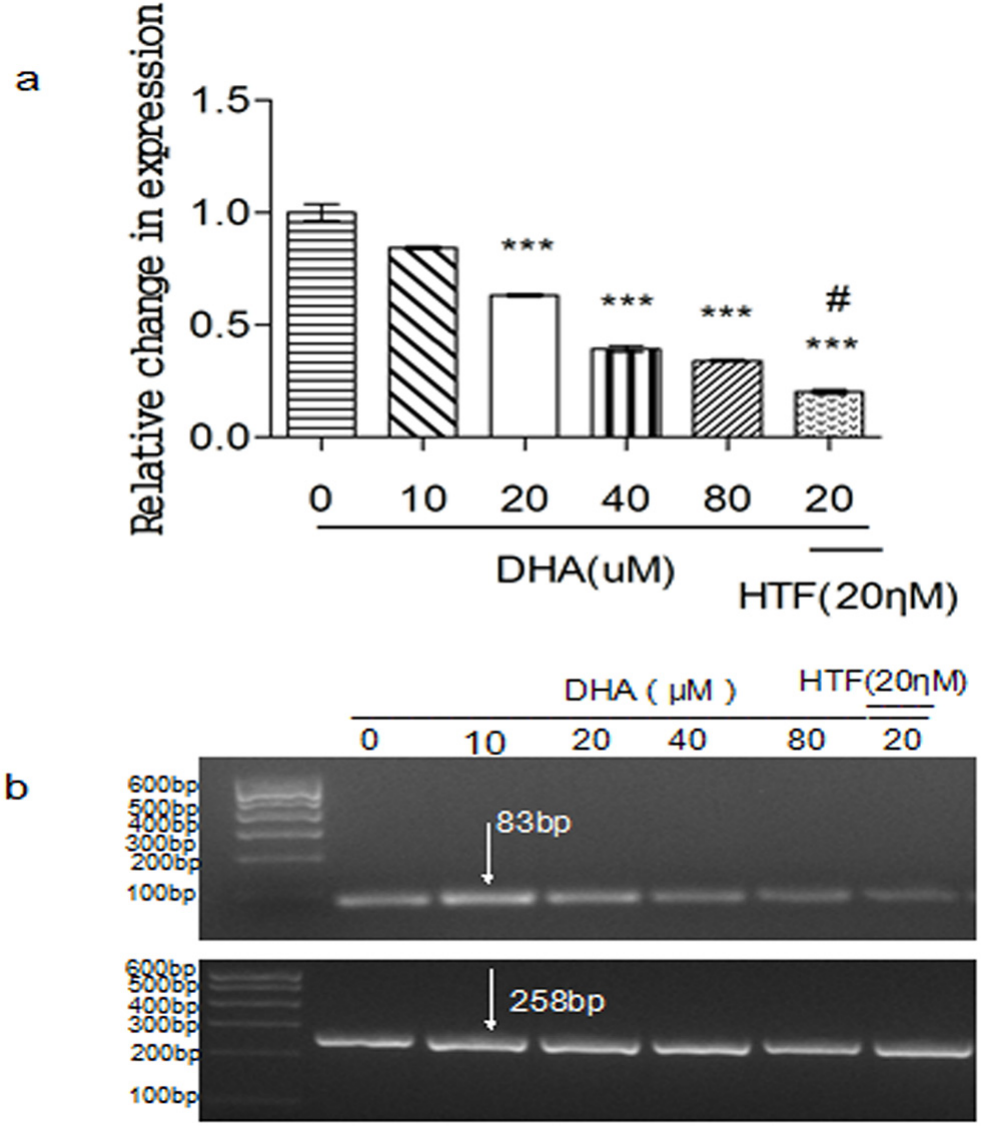
Treatment of Jurkat cells with DHA or DHA/HTF inhibited TfR mRNA expression. Jurkat cells (1 × 10^6^/well) were exposed to DHA (0, 10, 20, 40, 80 μM) or DHA/HTF for 48 h, Treatment of Jurkat cells with DHA induced a dose-dependent decrease in TfR expression (a). Compared with DHA treatment, DHA/HTF treatment decreased TfR expression by 43.21% (p < 0.001). Agarose gel electrophoresis showed that PCR products were of the expected size (b). GAPDH: 258 bp; TfR: 83 bp. The results represented mean ± standard deviation. ***p<0.001, compared with the control group, **#** p<0.001, compared with DHA.

Treatment of Jurkat cells with DHA induced a dose-dependent decrease in TfR expression (Fig 5A). TfR expression levels in cells treated with DHA/HTF or 20 μM DHA were 20.2% and 63.4% that of control, respectively, and the difference was statistically significant (p < 0.001). Agarose gel electrophoresis showed that PCR products were of the expected size (Fig 5B).

### Treatment of Jurkat cells with DHA or DHA/HTF inhibited VEGF mRNA expression

**Fig 6:**
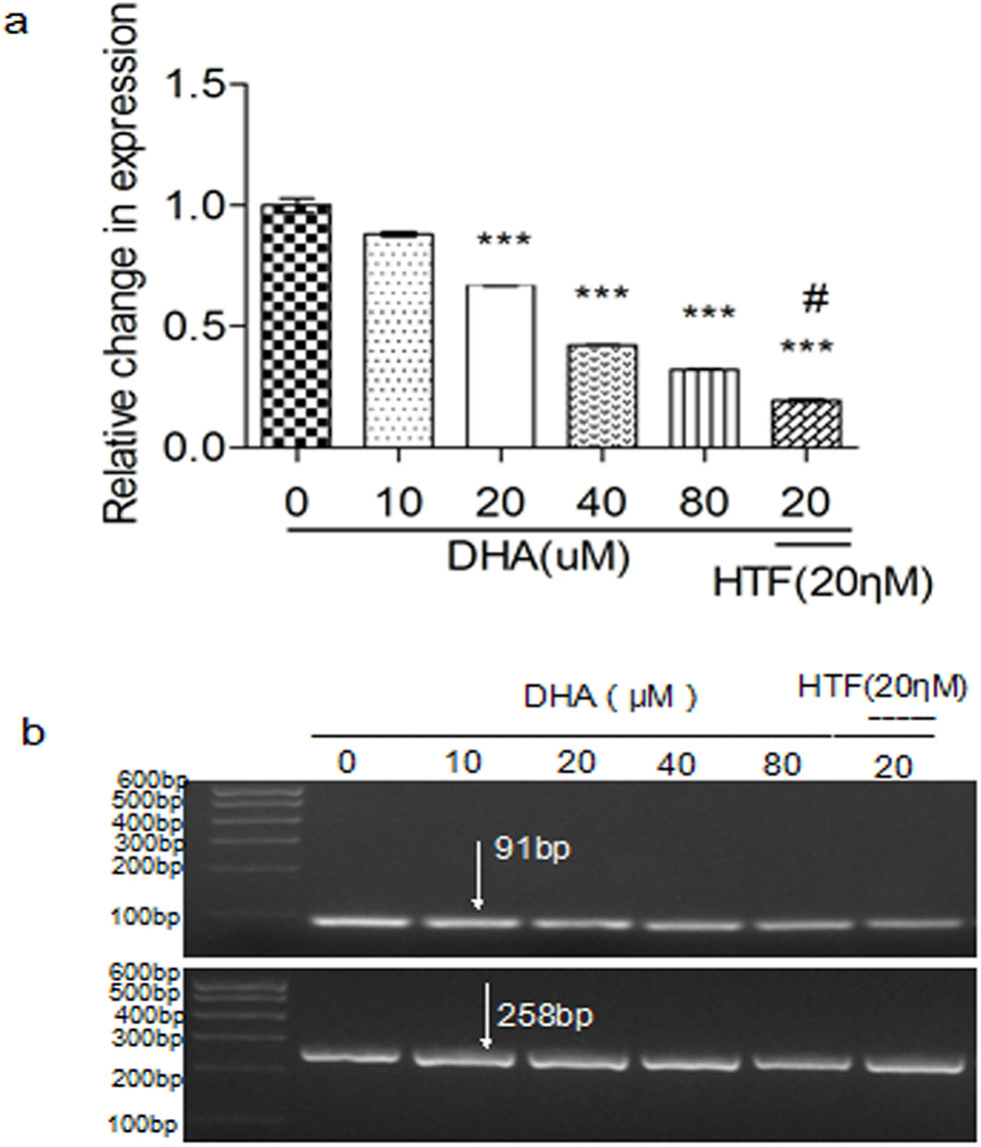
Treatment of Jurkat cells with DHA or DHA/HTF inhibited VEGF mRNA expression. Jurkat cells (1 × 10^6^/well) were exposed to DHA (0, 10, 20, 40, 80 μM) or DHA/HTF for 48 h, treatment of Jurkat cells with DHA induced a dose-dependent decrease in VEGF expression (a). Compared with DHA treatment, DHA/HTF treatment decreased VEGF expression by 47.77% (p < 0.001). Agarose gel electrophoresis showed that PCR products were of the expected size (b). GAPDH: 258 bp; VEGF: 91 bp. The results represented mean ± standard deviation. ***p<0.001, compared with the control group, **#** p<0.001, compared with DHA.

Treatment of Jurkat cells with DHA induced a dose-dependent decrease in VEGF expression (Fig 6A). VEGF expression levels in cells treated with DHA/HTF or 20 μM DHA were 19.4% and 67.2% that of the control, respectively, and the difference was statistically significant (p < 0.001). Agarose gel electrophoresis showed that PCR products were of the expected size (Fig 6B).

### Treatment of Jurkat cells with DHA or DHA/HTF inhibited hTERT expression

**Fig 7:**
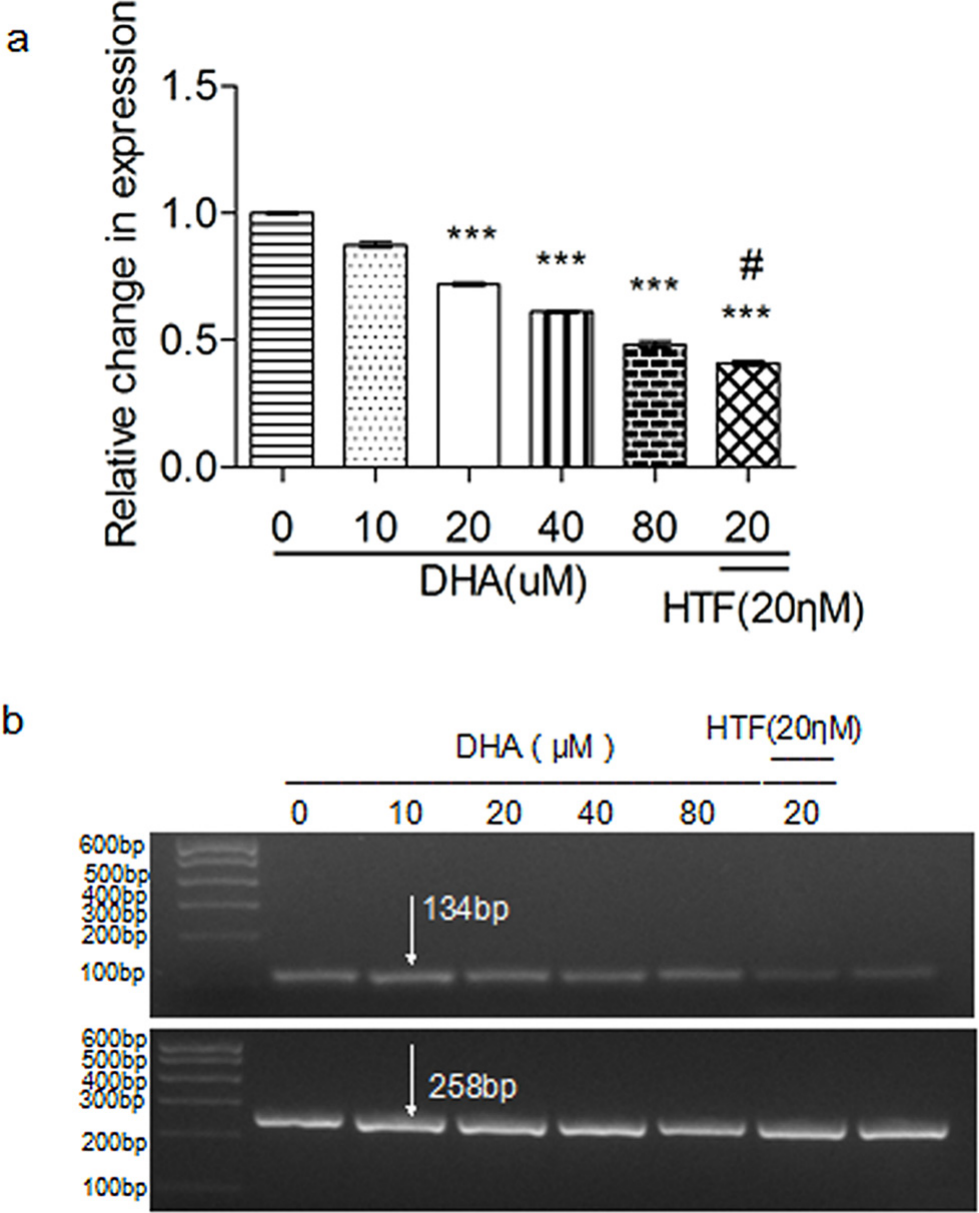
Treatment of Jurkat cells with DHA or DHA/HTF inhibited telomerase activity. Jurkat cells (1 × 10^6^/well) were exposed to DHA (0, 10, 20, 40, 80 μM) or DHA/HTF for 48 h, treatment of Jurkat cells with DHA induced a dose-dependent decrease hTERT expression (a). Compared with DHA treatment, DHA/HTF treatment decreased hTERT expression by 31.13% (p < 0.001). Agarose gel electrophoresis showed that PCR products were of the expected size (b). GAPDH: 258 bp; hTERT: 134 bp. The results represented mean ± standard deviation. ***p<0.001, compared with the control group, **#** p<0.001, compared with DHA.

Treatment of Jurkat cells with DHA induced a dose-dependent decrease of hTERT expression (Fig 7A). hTERT expression levels in cells treated with 20 μM DHA or DHA/HTF were 72.0% and 40.9% that of the control, respectively, and the difference was statistically significant (p < 0.001). Agarose gel electrophoresis showed that PCR products were of the expected size (Fig 7B).

## Discussion

In the current work, we examined the cytotoxic effects and antineoplastic mechanisms underlying DHA- and DHA/HTF-mediated inhibition of T-cell lymphoma. DHA decreased Jurkat cell viability in a dose- and time-dependent manner, with the IC50 value of DHA combined with HTF was 3.4 times lower than DHA alone. The antineoplastic effect of DHA was also enhanced by HTF. These results are consistent with at least two other studies [25, 26]. These results indicate that some cells, including T-cell lymphoma cells and retinoblastoma cells, are susceptible to DHA, and indicate that combination with HTF can increase the cytotoxicity of DHA.

Apoptosis is the primary mechanism responsible for the antineoplastic effect of DHA. In our study, apoptosis of Jurkat cells increased with DHA treatment in a dose-dependent manner. Previous studies in prostate cancer and K562 cells are consistent with our findings [26, 27]. Furthermore, we found that cells treated with DHA/HTF had a 2.75-times higher rate of apoptosis than cells treated with DHA alone.

Killing of T-cell lymphoma cells is associated with increased generation of ROS, and apoptosis is associated with ROS production [28, 29]. The peroxide bridge within the DHA molecule can react with iron to produce ROS, leading to macromolecular damage and cell death [9, 12]. We found that ROS production in DHA/HTF-treated cells was 1.66 times higher than that of cells treated with DHA alone. Moreover, ROS generation increased as the concentration of DHA increased. These results indicate that the cytotoxic effects of DHA and DHA/HTF on T-cell lymphoma cells were associated with ROS generation and cell apoptosis.

We also found that DHA cytotoxicity impacted cell cycle progression, consistent with a prior report [30]. Our cell cycle analysis results revealed that DHA impaired T-cell lymphoma cell growth at the G0/G1 phase.

TfR is implicated in iron absorption and cell growth [31], and tumor cells can have high levels of TfR expression [18, 19]. We found that DHA treatment decreased TfR expression in a dose-dependent manner. Furthermore, co-treatment with HTF increased DHA-mediated TfR downregulation three-fold. Based on the above study, this allows us to propose that the anticancer mechanism underlying the significantly increased anticancer activity of DHA used in combination with HTF is that DHA is activated by HTF-mediated electrocatalytic reduction [32].

Intravasation and metastasis of tumor cells requires tumor angiogenesis. An anti-angiogenic effect of DHA has been described [33, 34], and VEGF is a very effective angiogenic factor. We found that DHA treatment of T-cell lymphoma cells decreased VEGF expression in a dose-dependent manner. Furthermore, compared with DHA treatment, VEGF expression decreased by DHA/HTF treatment. Our data indicate that DHA promotes an anti-angiogenic effect on lymphoma, which is enhanced by combination with HTF.

The development of lymphoma has also been associated with abnormal activation of telomerase. Telomerase activity is significantly increased in patients with lymphoma. [35] We found that DHA treatment decreased Jurkat cell hTERT expression in a dose-dependent manner. Compared with DHA treatment, hTERT expression decreased by DHA/HTF treatment. These results indicate that DHA/HTF could significantly impair telomerase activity, facilitating the anti-proliferative effects of DHA on T-cell lymphoma cells. However, the potential mechanisms of telomerase activity impaired by DHA/HTF will need to be explored in further investigations.

Together our results demonstrate that DHA is cytotoxic to T-cell lymphoma cells, and this effect is enhanced by co-treatment with HTF. DHA also induced ROS generation, downregulated TfR, VEGF, and hTERT mRNA expression, promoted apoptosis, and triggered cell cycle arrest. These results suggest that DHA may be a useful therapeutic agent for T-cell lymphoma patients. Our results contribute to a better understanding of the effects of DHA/HTF on T-cell lymphoma cells. Taken together, our data reveal an antineoplastic effect of DHA and DHA/HTF on T-cell lymphoma and the mechanisms underlying this effect.
